# Systematic re-evaluation of the long-used standard protocol of urease-dependent metabolome sample preparation

**DOI:** 10.1371/journal.pone.0230072

**Published:** 2020-03-17

**Authors:** Jungyeon Kim, Joong Kyong Ahn, Yu Eun Cheong, Sung-Joon Lee, Hoon-Suk Cha, Kyoung Heon Kim

**Affiliations:** 1 Department of Biotechnology, Graduate School, Korea University, Seoul, Republic of Korea; 2 Division of Rheumatology, Department of Internal Medicine, Kangbuk Samsung Hospital, Sungkyunkwan University School of Medicine, Seoul, Republic of Korea; 3 Division of Rheumatology, Department of Medicine, Samsung Medical Center, Sungkyunkwan University School of Medicine, Seoul, Republic of Korea; Helen DeVos Children’s Hospital, UNITED STATES

## Abstract

In the urinary metabolomics for finding biomarkers in urine, owing to high concentrations of urea, for chromatography-based metabolomic analysis, urea needed to be degraded by urease. This urease pretreatment has been the key step of sample preparation for standard urinary metabolomics until today even for mass spectrometry-based analysis. The urease pretreatment involving incubation of urine with urease contradicts the concept of metabolome sampling, which should immediately arrest metabolic reactions to prevent alterations of a metabolite profile. Nonetheless, the impact of urease pretreatment has not been clearly elucidated yet. We found that activities of urease and endogenous urinary enzymes and metabolite contaminants from the urease preparations introduce artefacts into metabolite profiles, thus leading to misinterpretation.

## Introduction

Urine is one of the most commonly used biological matrices for clinical tests owing to noninvasive and convenient collection and the presence of abundant metabolites reflecting biological and metabolic status [1–7]. Most of metabolic diseases such as gout and diabetes alter metabolism, thus resulting in changes in urinary metabolites [8,9]. The study on global changes of metabolite profiles of living organisms and biological matrices such as blood and urine is called metabolomics [10,11], and this approach is frequently exploited to find biomarkers and unknown pathology in diseases [2,3,7].

In metabolomics, to obtain accurate, reproducible, and reliable metabolome data, optimized standard protocols are needed for metabolome sample preparation [11–14]. For the urinary metabolomics based on a chromatography–mass spectrometry system, a standard protocol has been suggested and widely used [2], and this standard protocol includes collecting urine samples; storage below −20°C; urease pretreatment for degrading urea using a plant urease from jack beans [2,15]; extraction of metabolites; precipitation of DNA, RNA, and proteins with methanol; analysis of metabolites using a chromatography–mass spectrometry system; and biological interpretation based on statistical analyses [2]. On the other hand, the urease pretreatment step is likely to alter metabolite profiles, thereby leading to misinterpretation of metabolomic data. This is because urease pretreatment involving incubation with the enzyme ironically contradicts the standard metabolome sample preparation concept, according to which arresting any metabolic reactions and minimizing artefacts are key to obtaining representative and reproducible metabolite profiles [10,13,14,16]. In addition, even in clinical laboratories, urinalysis is performed immediately after sampling to avoid possible metabolic alterations in urine samples [1,17]. Therefore, controversy has been raised about the standard protocol of sample preparation for urinary metabolomics based on a chromatography–mass spectrometry system [18,19].

During urinary metabolome sample preparation, pretreatment of urine with urease is the first step. Urease pretreatment was introduced in the 1960s to degrade urea into ammonia and CO_2_ in urine samples when chromatography was conducted alone to analyze urinary metabolites [20–23]. The original purposes of removing urea from urinary metabolome samples were to minimize severe disturbances of chromatographic analysis [24–26], possible incomplete derivatization [19,20], and the risk of column overloading [27], owing to high concentrations of urea in urine. Since then, urinary metabolomic tools advanced to mass spectrometry-based analyses, in which metabolites can be analyzed and identified by means of mass spectra, despite overlapping peaks in chromatograms [18]. Therefore, urease pretreatment became an unessential step for urine metabolomics, but most urinary metabolomic studies follow the standard protocol [2] and still include urease pretreatment [4–6,19,28]. Only a few urinary metabolomic analyses have been performed without urease pretreatment [18,29]. Although significant changes of metabolite profiles were observed after urease pretreatment [18,19], actual causes of changes in metabolite profiles have not been suggested. Webb-Robertson et al. [19], showed that metabolite changes occur when urine samples are incubated with or without urease. However, they did not consider the cause of the changes and suggested that the benefits of urease pretreatment outweighed the adverse effects of potential artefacts. Kind et al. [18] has observed decreases in the intensity of certain metabolites after the urease pretreatment. However, they did not consider the causes of the changes and removed the urease pretreatment from the urine metabolite preparation steps. Therefore, systematic evaluation of the effects of urease pretreatment on metabolite profiles and assessment of the necessity of urease pretreatment for urinary metabolomics are necessary to decide whether or not to keep the urease pretreatment.

The second step of urinary metabolome sample preparation is extraction of urinary metabolites with a solvent. To obtain representative and reproducible metabolome samples, the extraction solvent should be carefully selected so that it can ensure high performance on the effectiveness and reproducibility of metabolite extraction and effectiveness of precipitation of DNA, RNA, and proteins, which could be sources of artefacts in metabolite profiles and may interfere with metabolite derivatization [2,12–14,16]. In the standard protocol of urinary metabolome sample preparation [2] and most urinary metabolomic studies [4–6], methanol is the most common solvent. Ethanol is also frequently used for urinary metabolite extraction [30]. Other solvents such as a mixture of acetonitrile and water; a mixture of water, isopropanol, and methanol; and acidified methanol are frequently employed for metabolome extraction of various biological tissues and organisms [11,12,31]. Although optimization and evaluation of extraction solvents have been performed for various organisms and biological samples [13,14,16], for urinary metabolite extraction, solvents have not been systematically evaluated and recommended yet.

The controversial urease pretreatment has long been used as an essential step in the standard urinary metabolome sample preparation. In this study, the effects of urease pretreatment on metabolite profiles and the necessity of urease pretreatment were investigated along with the systematic evaluation of solvents for urinary metabolite extraction. To accomplish these tasks, metabolites were extracted from 68 human urine samples, and gas chromatography with time-of-flight mass spectrometry (GC/TOF–MS) was carried out for identification and quantification of metabolites. These results appear to resolve the controversy regarding the necessity of urease pretreatment and seem to help to establish a more reliable standard protocol for urinary metabolome sample preparation.

## Materials and methods

### Collection of urine samples

A total of 68 human urine samples from healthy volunteers (31 males and 37 females) at ages in the 30s to 60s were obtained from the Samsung Medical Center in Seoul, South Korea (S1 Table). Following the standard sterile procedures, midstream urine samples were collected from all the fasting volunteers in the morning. Urine samples were centrifuged at 16,100 × *g* and 4°C for 30 min, and then supernatants were immediately frozen and stored at −80°C. The experimental protocols used in this study were approved by the Institutional Review Boards of Samsung Medical Center (2018-03-147-001) and Kangbuk Samsung Hospital (KBSMC2013-01-023), and informed consent for the enrollment in this study was provided by the volunteers. This study was conducted in accordance with the Declaration of Helsinki.

### Preparation of pooled urine samples with or without thermal treatment

Sixty-eight urine samples as 1 ml aliquots were mixed to make a urine pool. To inactivate endogenous urinary enzymes by thermal treatment, a half of the urine pool was autoclaved at 121°C for 15 min. To prevent any possible changes in metabolites, urinary metabolites were immediately extracted from pooled urine samples which were thermally not treated (NT) or thermally treated (TT). Each entire procedure was independently repeated six times.

### Preparation of urinary metabolite extracts depending on urease pretreatment

Urine samples from individuals or pooled urine samples were thawed on ice for 120 min and vortexed for 1 min. To extract metabolites from urine samples incubated with urease, 100 μl of each urine sample was mixed with 10 μl of urease, which is equivalent to 100 U of Type 3 urease (U1500; Sigma-Aldrich, St. Louis, MO), and the mixture was incubated at 37°C for 60 min. After that, 890 μl of methanol was added to the mixture, the latter was thoroughly vortexed for 5 min and centrifuged at 16,100 × *g* and 4°C for 10 min. From the supernatant, 400 μl was collected, vacuum-dried in a vacuum concentrator (Labconco, Kansas City, MO), and stored at −80°C until further analysis to prevent the changes in metabolites. Each entire procedure was independently repeated six times.

To extract metabolites from pooled urine samples incubated with water without urease, 100 μl of individual urine samples was mixed with 10 μl of distilled water, and incubated at 37°C for 60 min. To directly extract metabolites from pooled urine samples without incubation, 100 μl of individual urine samples was mixed with 10 μl of distilled water. The rest of the experimental procedure was the same as that for the urine samples incubated with urease. Each entire procedure was independently repeated six times.

### Preparation of urinary metabolite samples using the two types of ureases

To study the introduction of metabolite contaminants from urease preparations into urinary metabolome samples during urease pretreatment, 0, 0.001, 0.01, 0.1, 1, 10, and 20 μl of a solution of Type 3 urease (U1500; Sigma-Aldrich) or Type 4 urease (U4200; Sigma-Aldrich), which were equivalent to 0, 0.01, 0.1, 1, 10, 100, and 200 U of urease, were mixed with 100 μl of distilled water and incubated at 37°C for 60 min. After that, 890 μl of methanol was added to the mixture, and the latter was thoroughly vortexed for 5 min and centrifuged at 16,100 × *g* and 4°C for 10 min. Five hundred microliters of the supernatant, which was equivalent to 0, 0.005, 0.05, 0.5, 5, 50, and 100 U of the Type 3 or 4 urease, was collected, vacuum-dried, and stored at −80°C until further analysis. Each entire procedure was independently repeated eight times.

### Extraction of urinary metabolites with five extraction solvents

One hundred microliters of a thermally not treated urine pooled sample was mixed with 900 μl of different extraction solvents, 50ACN (acetonitrile:water at 1:1, v/v), AM (formic acid:methanol at 0.125:99.875, v/v), MeOH (pure methanol), WiPM (water:isopropanol:methanol at 2:2:5, v/v/v), and EtOH (pure ethanol) at −20°C. The mixture was thoroughly vortexed for 5 min, and centrifuged at 16,100 × *g* and 4°C for 10 min. From each supernatant, 400 μl was collected, vacuum-dried, and stored at −80°C until further analysis to prevent the changes in metabolites. Each entire procedure was independently repeated six times.

### A comparison of precipitating capabilities of the extraction solvents

One milliliter each of thermally not treated urine pooled samples was mixed at −20°C with 9 ml of one of the following extraction solvents: 50ACN, AM, MeOH, WiPM, and EtOH. The mixtures were thoroughly vortexed for 5 min, and centrifuged at 16,100 × *g* and 4°C for 10 min. The supernatants were removed, and precipitates were dried and weighed. Each entire procedure was independently repeated four times.

### Analysis of urinary metabolites by GC/TOF-MS

Before urinary metabolite analysis by GC/TOF-MS, metabolites were derivatized by methoximation and silylation. For the methoximation, metabolite samples were incubated with 10 μl of 40 mg/ml methoxyamine hydrochloride (Sigma-Aldrich) in pyridine at 30°C for 90 min. The methoximated samples were then incubated with 50 μl of *N*-methyl-*N*-trimethylsilyl-trifluoroacetamide (Fluka, Buchs, Switzerland) at 37°C for 30 min for the silylation. Then, 2 μl of a mixture of authentic standard methyl esters of C8, C9, C10, C12, C14, C16, C18, C20, C22, C24, C26, C28, and C30 fatty acids (Sigma-Aldrich) was added to the derivatized metabolite samples as retention index markers.

For identification and relative quantification of metabolites, an Agilent 7890B GC (Agilent Technologies, Santa Clara, CA) coupled with a Pegasus HT-TOF MS (LECO, St. Joseph, MI) was used. A derivatized metabolite sample (0.5 μL) was injected into the GC in splitless mode. The injected metabolite sample was separated on an RTX-5Sil MS column (30 m length, 0.25 mm inner diameter, and 0.25 μm film thickness; Restek, Bellefonte, PA) with a 10 m guard column. Oven temperature was set to 50°C for 1 min, ramped to 330°C at a rate of 20°C/min, and held at 330°C for 5 min. Mass spectra were recorded in the *m/z* range of 85–500 at an acquisition rate of 10 spectra/s. The ion source and transfer line temperatures were set to 250 and 280°C, respectively, and the ionization by electron impact was performed at 70 eV.

For accurate analysis of metabolites by GC/TOF-MS without external interference, daily quality control was carried out. Two blank method samples and four calibration curve samples consisting of 31 pure reference compounds including amino acids, organic acids, and sugars were all derivatized by the same procedures as those used for the urinary metabolite analysis described above [32]. To avoid batch effects, samples in each experiment were randomly ordered and then analyzed all at once. To avoid the effects of impurities in the solvent, pure acetonitrile was analyzed via the same protocol once every 10 samples.

### Data processing for GC/TOF-MS and statistical analyses

For detection and deconvolution of mass spectra, LECO Chroma TOF software (C version; LECO) was used for preprocessing of mass spectral data. The preprocessed data were next processed by means of the BinBase in-house software [33]. BinBase identified peaks by referring mass spectra and retention indices of peaks to Fiehn, NIST, and in-house libraries [33,34]. The peaks with mass spectral similarity above 700 with the authentic standards were considered identified metabolites. Quantities of the identified metabolites were reported as peak heights of their unique ion intensities. For each positively detected spectrum, the lowest background intensity was subtracted from the intensity of the quantified ion in its retention time region ± 5 s using the MZmine software [33].

Raw peak intensities without any normalization or transformation were subjected to multivariate and nonparametric statistical analyses, PCA, PLS-DA, the Mann–Whitney *U* test, the Wilcoxon signed-rank test, the Kruskal–Wallis test followed by *post hoc* Mann–Whitney *U* test with FDR adjusted via the Benjamini–Hochberg correction, heat map construction, and the MetaMapp analysis. PCA and PLS-DA were performed in the SIMCA-P+ software (version 12.0; Umetrics AB, Umea, Sweden), and the heat map was visualized in the MultiExperiment Viewer application [35]. The Mann–Whitney U test, Wilcoxon signed-rank test, and Kruskal–Wallis test were performed in Metabox [36]. The MetaMapp analysis was performed in MetaMapp and Cytoscape software packages [37].

## Results

### Effects of urease pretreatment on urinary metabolite profiles

In this study, we analyzed 68 urine samples collected from 31 healthy males and 37 healthy females (S1 Table). To investigate possible changes and alterations of urinary metabolites after pretreatment of urine with urease, urine metabolome samples were prepared by three pretreatment methods. First, according to the standard protocol [2], urine samples were incubated with urease and extracted with a solvent, pure methanol (Group UI), where urease pretreatment is the key step of the standard protocol of urine metabolome sample preparation. Second, to investigate the effects of urease pretreatment, urine samples were incubated with water without urease and extracted with pure methanol (Group WI). Third, to assess the effects of incubation at the urease pretreatment temperature, urine samples were directly extracted with pure methanol without urease pretreatment (Group DE). With all the different pretreatment methods, a total of 107 metabolites were identified by GC/TOF–MS in extracted metabolome samples (S3 Table).

**Fig 1:**
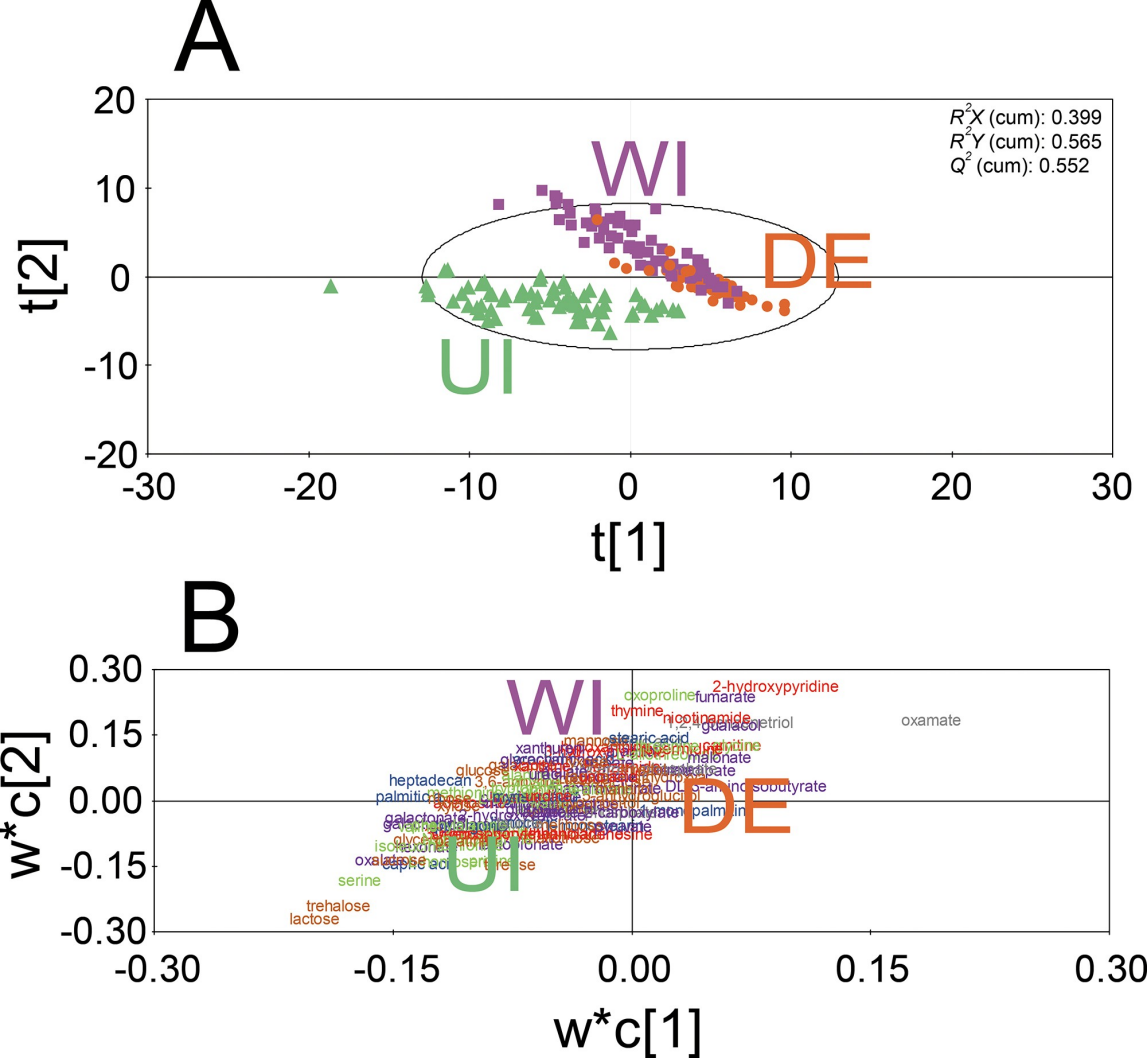
PLS-DA and a heat map of metabolite profiles of urinary metabolome samples obtained from 68 healthy individuals by different pretreatment methods. (A) A score plot and (B) loading plot of the PLS-DA model of 106 identified metabolites excluding urea. The urinary metabolome samples were prepared as follows: group UI, incubated with urease and extracted with pure methanol; group WI, incubated with water without urease and extracted with pure methanol; group DE, extracted using pure methanol without urease pretreatment. The color of each metabolite indicates the chemical class (amine; red, fatty acid; blue, organic acid; purple, sugar and sugar alcohols; orange, miscellaneous; gray) of the metabolite.

To find any differences in metabolite profiles among urinary metabolome samples subjected to the three different pretreatment methods, a multivariate analysis, i.e., partial least-squares discriminant analysis (PLS-DA), was performed on the 106 identified metabolites excluding urea (Fig 1A and 1B). The score plot (Fig 1A) and loading plot (Fig 1B) of the PLS-DA model showed complete separation of group UI from the other groups. Groups DE and WI were separated partially. The PLS-DA model showed high fitness [*R*^*2*^*X* (cumulative) of 0.369 and *R*^*2*^*Y* (cumulative) of 0.565] and a high predictive ability: *Q*^*2*^ (cumulative) of 0.552.

To find significant changes in metabolite abundance depending on the pretreatment methods, the Kruskal–Wallis test with the *post hoc* U test was performed. As a result, the abundance levels of 103 metabolites were found to significantly change (false discovery rate [FDR]-adjusted *p* value <0.05; S4 Table). In a comparison of groups UI and WI, 53 metabolites were significantly more abundant in group UI, and 16 metabolites were significantly more abundant in group WI. In a comparison of groups UI and DE, 87 metabolites were significantly more abundant in group UI, and six metabolites significantly more abundant in group DE. In a comparison of groups DE and WI, 92 metabolites were significantly more abundant in group DE, and only one metabolite was significantly more abundant in group WI.

### Effects of urease pretreatment on the gender-discriminating ability of the urinary metabolome

**Fig 2:**
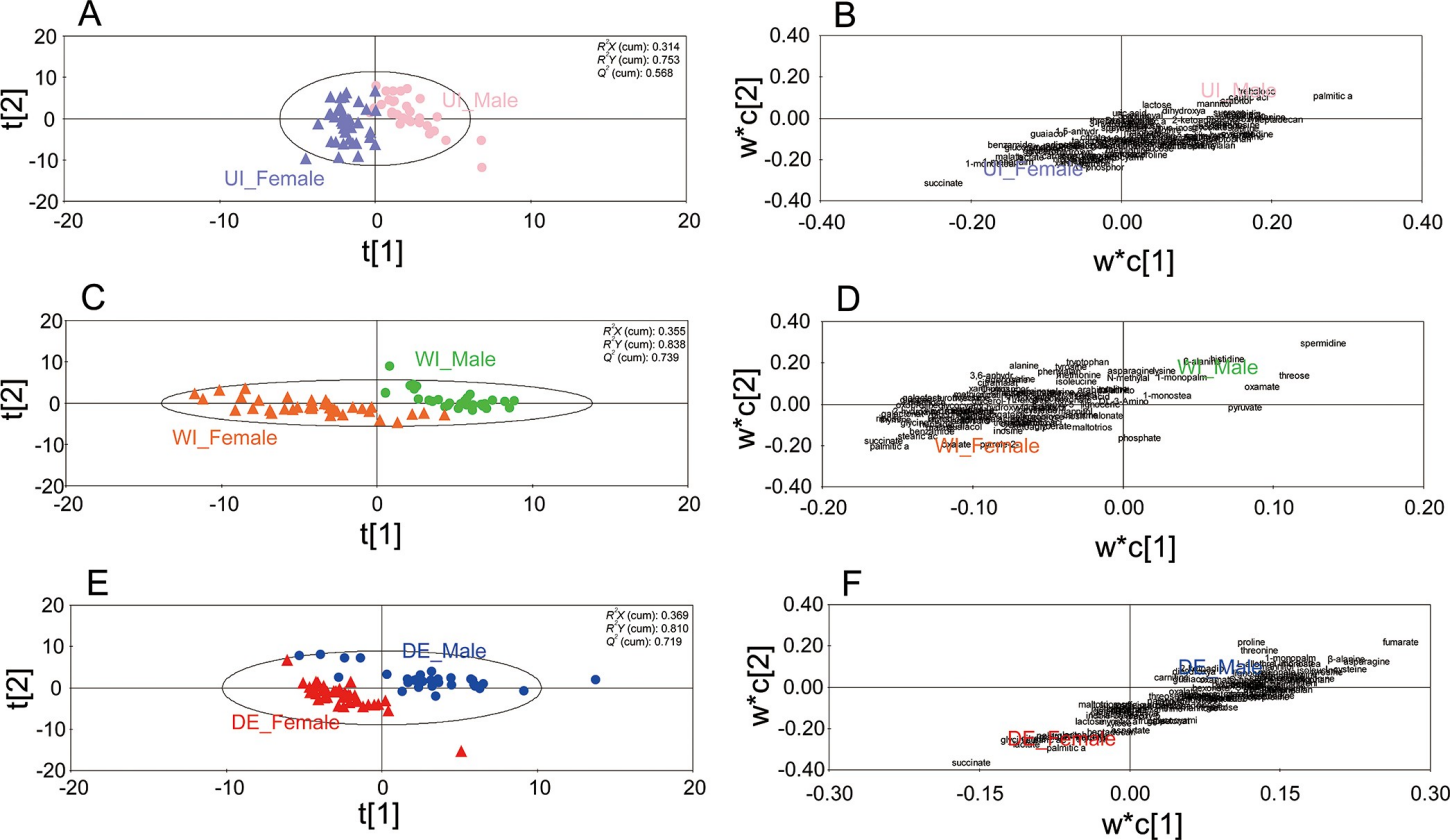
PLS-DA scores plots (A,C,E) and loading plots (B,D,F) of metabolite profiles for 106 metabolites (excluding urea) identified in urinary metabolome samples obtained from 31 male and 37 female individuals by the following pretreatment methods. UI_Male, the male urine sample group incubated with urease and extracted with pure methanol; UI_Female, the female urine sample group incubated with urease and extracted with pure methanol; WI_Male, the male urine sample group incubated with water without urease and extracted with pure methanol; WI_Female, the female urine sample group incubated with water without urease and extracted with pure methanol; DE_Male, the male urine sample group extracted using pure methanol without urease pretreatment; DE_Female, the female urine sample group extracted by means of pure methanol without urease pretreatment.

Urine is known to uniquely contain gender-discriminating biomarkers [19,38,39]. In this study, it was tested how urine pretreatment affects the gender-discriminating ability of the urinary metabolome. To this end, PLS-DA models of 106 metabolites prepared by the three urine pretreatment methods (UI, WI, and DE) were set up for individual urine samples of males and females. Among the three urine pretreatment methods, group UI showed an overlap of urine samples between the genders. Accordingly, the urease-pretreated group (UI) showed the lowest discriminating ability: the lowest values of *R*^*2*^*X* (cumulative 0.314), *R*^*2*^*Y* (cumulative 0.753), and *Q*^*2*^ (cumulative 0.568; Fig 2A and 2B). In contrast, group WI showed complete separation of males and females (Fig 2C and 2D). Accordingly, group WI showed the highest discriminating ability among the three groups, namely, the highest values of *R*^*2*^*X* (0.355), *R*^*2*^*Y* (0.838), and *Q*^*2*^ (0.739). Group DE also showed complete separation of males and females (Fig 2E and 2F). Accordingly, group DE showed the second highest discriminating ability, i.e., high values of *R*^*2*^*X* (0.369), *R*^*2*^*Y* (0.810), and *Q*^*2*^ (0.719). The variable importance in projection (VIP) values of metabolites in the PLS-DA models are listed in S5 Table. Overall, the urease pretreatment was found to significantly reduce the gender-discriminating ability of urinary metabolomics.

### Effects of endogenous urinary enzymes on urinary metabolite profiles

According to the standard sample preparation protocol for urinary metabolomics [2], during urine pretreatment with urease, urine samples are incubated at 37°C to induce the enzymatic reaction driven by urease. Because endogenous urinary enzymes maintain their activities in urine after sample collection [40,41], not only added urease but also endogenous urinary enzymes present in urine may act on urinary metabolites during the incubation process after sample collection. In this study, possible effects of urinary enzymes on metabolite profiles during the incubation process were investigated. For this purpose, 68 urine samples were pooled together, and the pooled samples were thermally not treated (NT) or thermally treated (TT) to inactivate endogenous urinary enzymes in urine (Table 1). Metabolome samples from these two urine sample groups were next pretreated in one of the following two ways: i) incubated with water without urease and then extracted with pure methanol (WI); ii) directly extracted with the solvent without urease pretreatment (DE). Therefore, the following four urinary metabolome sample groups were established: combining NT and WI (group NT&WI), combining NT and DE (group NT&DE), combining TT and WI (group TT&WI), and combining TT and DE (group TT&DE). In the GC/TOF–MS analysis of these pooled metabolome samples, a total of 113 metabolites were identified (S6 Table).

**Table 1:** The study design regarding the effect of endogenous enzymes.

Sample	Enzyme inactivation	Group	Remarks
Urine pool from 68 healthy volunteers	Thermally not treated	Extraction after incubation with water (NT&WI; n = 6)	Changes represent activities from urinary enzyme
		Direct extraction (NT&DI; n = 6)	
	Thermally treated	Extraction after incubation with water (TT&WI; n = 6)	Changes represent experimental artefacts
		Direct extraction (TT&DI; n = 6)	

To compare metabolite profiles of the four groups—NT&WI, NT&DE, TT&WI, and TT&DE—principal component analysis (PCA) was performed (S1A and S1B Fig). The thermally not treated sample groups (NT), NT&WI and NT&DE, were completely separated from each other. On the other hand, in the thermally treated sample groups (TT)—TT&WI and TT&DE—the PCA model showed similar metabolite profiles regardless of WI or DE. The PCA model showed a high explanatory value of *R*^*2*^*X* (cumulative 0.705) and a high predictive value of *Q*^*2*^ (cumulative 0.640; S2A and S2B Fig).

**Fig 3:**
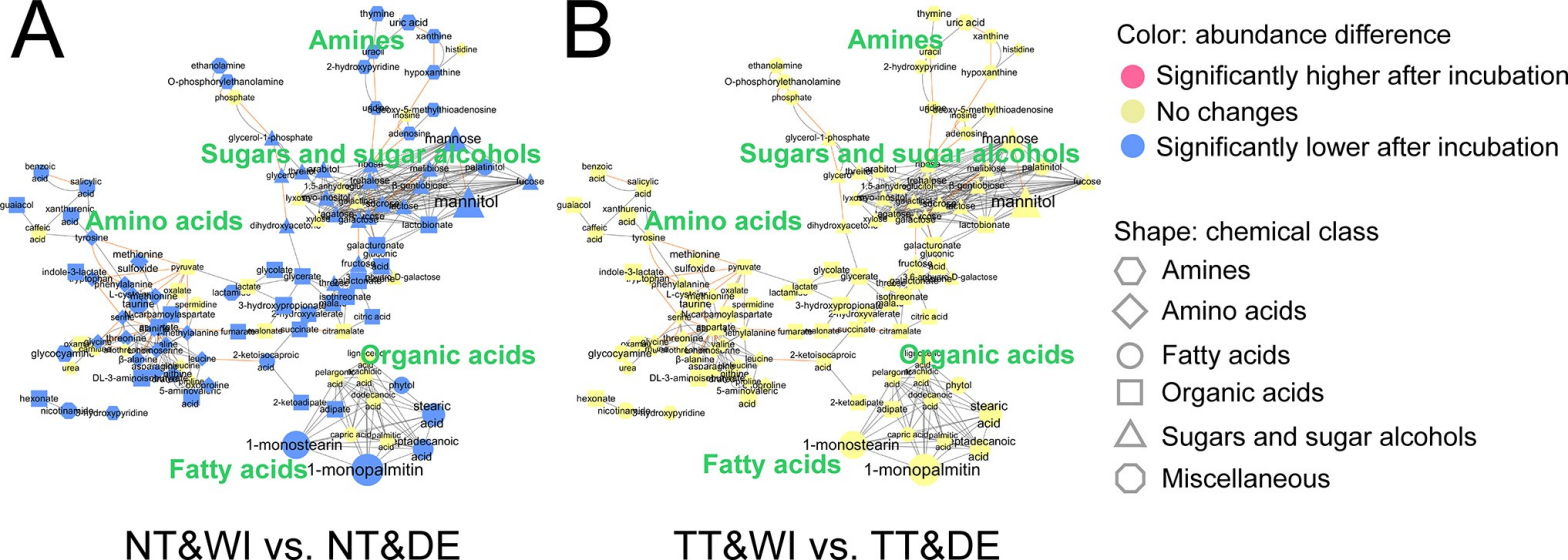
MetaMapp analysis of the effects of water incubation on the profiles of 113 metabolites identified in pooled urinary metabolome samples which were (A) thermally not treated or (B) thermally treated. Group NT&WI: thermally not treated, incubated with water without urease, and extracted with pure methanol; group NT&DE: thermally not treated and extracted with pure methanol without urease pretreatment; group TT&WI: thermally treated, incubated with water without urease, and extracted with pure methanol; group TT&DE: thermally treated and extracted with pure methanol without urease pretreatment. Classes of metabolites are represented by shapes. Significant increases and decreases in the abundance of metabolites after incubation with water are indicated by colors (*p* < 0.05, based on the Wilcoxon signed-rank test with FDR adjusted via the Benjamini–Hochberg correction). Magnitudes of fold changes are represented by the sizes of symbols and labels; biochemical and structural similarities are represented by orange and gray edges, respectively.

To study the effects of water incubation on metabolite profiles, groups NT&WI and NT&DE were compared, as were groups TT&WI and TT&DE. The comparisons were conducted by the Wilcoxon signed-rank test and visualized by means of MetaMapp (S7 Table, Fig 3). In MetaMapp (which visualizes differences in metabolite abundance), the thermally not treated groups (NT) showed the abundance levels of 87 metabolites to be significantly lower in group NT&WI than in group NT&DE (Fig 3A). In contrast, no significant differences were observed between groups TT&WI and TT&DE, which were both thermally treated to inactivate endogenous urinary enzymes (Fig 3B). In summary, endogenous urinary enzymes were found to significantly affect the metabolite profiles after incubation when urine samples were not thermally inactivated.

### Effects of urease itself on urinary metabolite profiles

**Fig 4:**
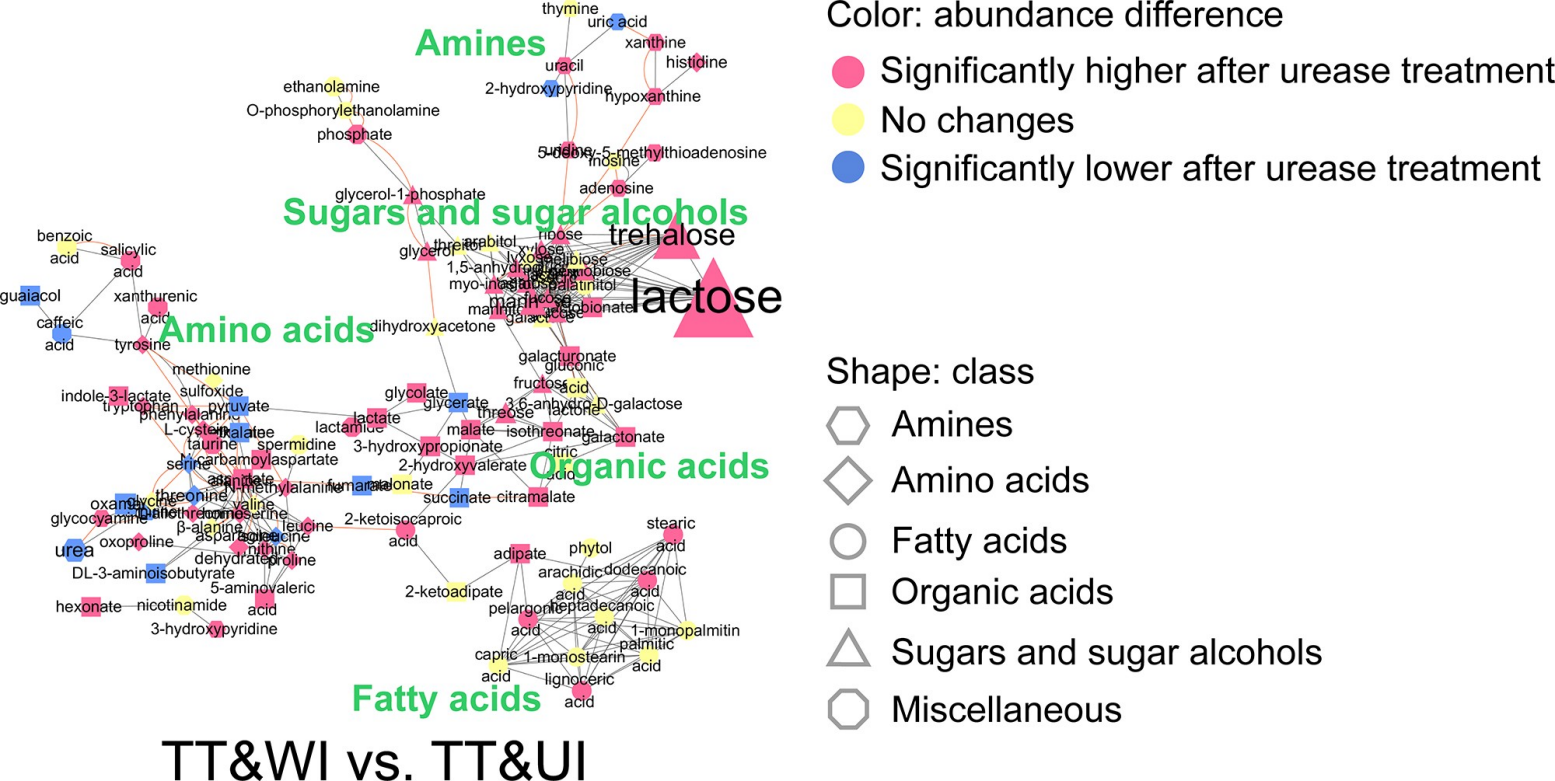
MetaMapp analysis of the effects of urease itself on the profiles of 113 metabolites identified in thermally treated pooled urinary metabolome samples. Group TT&WI: thermally treated, incubated with water without urease, and extracted with pure methanol; group TT&UI: thermally treated, incubated with urease, and extracted with pure methanol. Classes of metabolites are represented by shapes. Significant increases and decreases in the abundance of metabolites after incubation with urease are illustrated by colors (*p* < 0.05, based on the Mann–Whitney *U* test with FDR adjusted via the Benjamini–Hochberg correction). Magnitudes of fold changes are represented by the sizes of symbols and labels; biochemical and structural similarities are represented by orange and gray edges, respectively.

To study the effects of urease itself on the metabolite profiles of urine metabolome samples, the pooled and thermally treated urine samples were incubated with urease and extracted with pure methanol (group TT&UI). The metabolite profiles of group TT&UI were compared with those of the pooled, thermally treated, incubated with water, and pure methanol-extracted samples (group TT&WI) or the pooled, thermally treated, and directly pure methanol-extracted samples (group TT&DE) by PCA (S2A and S2B Fig). In the PCA model of the thermally treated groups (TT), the urease-treated group, TT&UI, was well separated from groups TT&WI and TT&DE, which were both not treated with urease (S2A Fig). The PCA model yielded a high explanatory value of *R*^*2*^*X* (0.688) and a high predictive value; the Mann–Whitney *U* test was performed, and the results were visualized in MetaMapp (S7 Table, Fig 4). Of the metabolites in group TT&UI, 66 metabolites were significantly more abundant and 16 metabolites significantly less abundant than their corresponding metabolites in group TT&WI.

**Fig 5:**
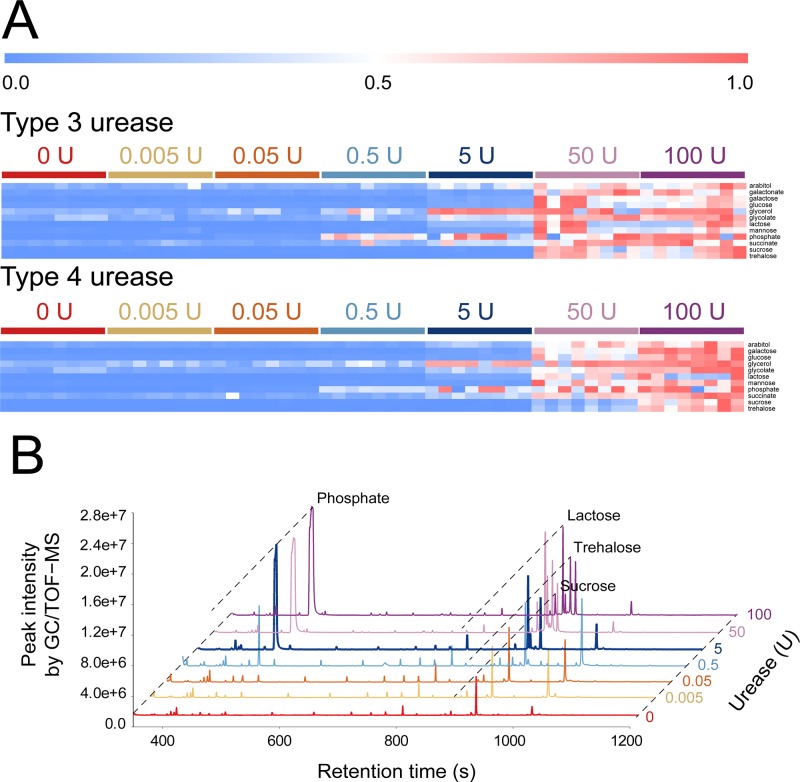
Increases in the abundance of metabolites in response to increasing the loading units (U) of Type 3 and Type 4 ureases. (A) A heat map of the 12 significantly upregulated metabolites that were derived from the Type 3 urease and the significantly upregulated 11 metabolites that were derived from the Type 4 urease, with an increase in the urease loadings (*p* < 0.05, according to the Kruskal–Wallis test with FDR adjusted via the Benjamini–Hochberg correction). (B) Total ion chromatograms for different loading units (U) of the Type 3 urease.

To investigate possible contamination by innate ingredients of urease preparations, Type 3 and Type 4 ureases, which are commonly used for urease pretreatment, were extracted with methanol, and these methanol extracts were analyzed. The Kruskal–Wallis test indicated that intensities of 12 metabolites (arabitol, galactonate, galactose, glucose, glycerol, glycolate, lactose, mannose, phosphate, succinate, sucrose, and trehalose) significantly increased as the loading amounts of the Type 3 urease in methanol extraction increased (S8A Table). In the methanol extract of the Type 4 urease, 11 metabolites (excluding galactonate), which increased in abundance with an increase in the loading amounts of the Type 3 urease during methanol extraction as mentioned above, significantly increased in abundance as the loading amounts of the Type 4 urease increased (S8B Table). The abundance levels of the upregulated metabolites were converted into relative abundance levels by dividing the abundance of each metabolite by the highest abundance of each metabolite across the samples, and these values were then visualized via a heat map (Fig 5A). The heat map revealed the abundance increases of 12 metabolites in the methanol extract of the Type 3 urease and the abundance increases of 11 metabolites in the methanol extract of the Type 4 urease, as the loading amounts of these ureases in the methanol extract of the ureases increased (Fig 5A). Among the upregulated metabolites, 10 metabolites (arabitol, galactonate, glucose, glycerol, glycolate, lactose, mannose, phosphate, sucrose, and trehalose) were also more abundant in group TT&UI than in group TT&WI (Fig 4). Peak intensities of phosphate and lactose, which showed the highest peak intensities among the upregulated metabolites in the methanol extracts of both ureases, reached saturation values when the loading amounts of both ureases increased to 5 units (Fig 5B).

### Evaluation of solvents on extraction of metabolites from urine

**Fig 6:**
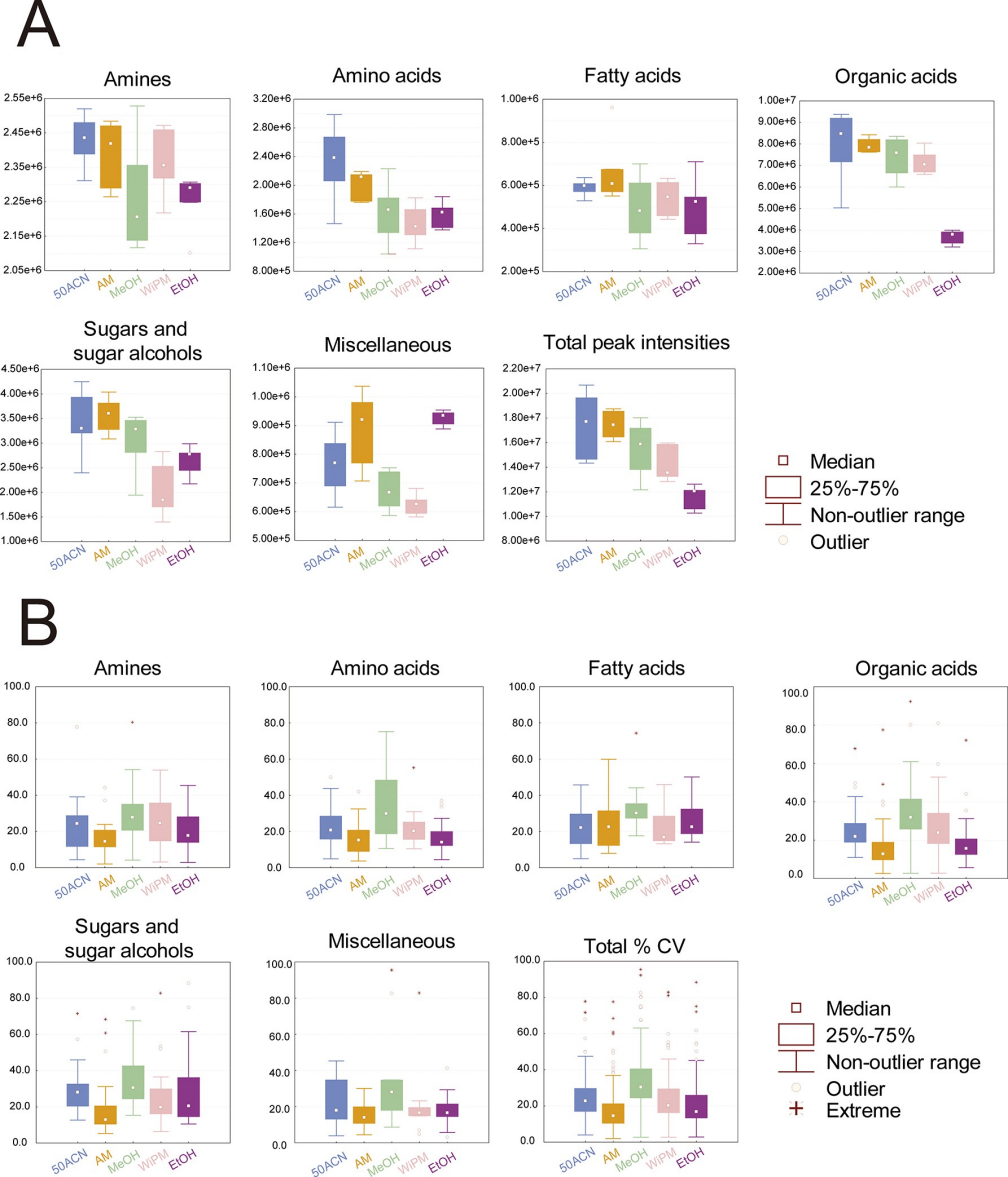
Box and whisker plots for the comparison of (A) the sum of peak intensities of the identified metabolites in each chemical class and (B) %CVs of peak intensities of the identified metabolites in each chemical class, in which the identified metabolites were extracted at −20°C from thermally not treated pooled urine samples by means of acetonitrile–water (50ACN; 1:1, v/v), formic acid–methanol (acidified methanol, AM; 0.125:99.875, v/v), pure methanol (MeOH), water–isopropanol–methanol mixture (WiPM; 2:2:5, v/v/v), or pure ethanol (EtOH).

To identify an ideal solvent for extracting urinary metabolites, five extraction solvents that possess distinctive solvent properties and are commonly used for metabolite extraction, namely, 50ACN (acetonitrile:water = 1:1, v/v), AM (acidified methanol, i.e., formic acid:methanol at 0.125:99.875, v/v), MeOH (pure methanol), WiPM (water:isopropanol:methanol at 2:2:5, v/v/v), and EtOH (pure ethanol), were evaluated in this study. These five solvents were applied to extraction of metabolites from thermally not treated pooled urine samples. To evaluate the metabolite extraction capabilities of the five extraction solvents, sums of identified peak intensities for each chemical class and the total for identified metabolites were compared using box and whisker plots (Fig 6A). Overall, solvents 50ACN and AM showed greater sums of peak intensities than did the other solvents. Especially in the classes of amines and fatty acids, 50ACN and AM both showed similarly greater sums of peak intensities than did those of other solvents. 50ACN manifested the largest sums of intensities in the classes of amino acids and organic acids. AM showed the largest sums of intensities in the classes of sugars and sugar alcohols and miscellaneous.

To evaluate metabolite extraction reproducibility of the five solvents, the percent coefficients of variance (%CVs) of metabolite abundance levels in each metabolite class were compared by means of box and whisker plots (Fig 6B). AM showed the lowest %CV in the classes of amines, amino acids, organic acids, sugars, and sugar alcohols as well as miscellaneous metabolites and in the total of identified metabolites compared with those of other solvents. Only the class of fatty acids showed similar %CVs among the extraction solvents.

**Fig 7:**
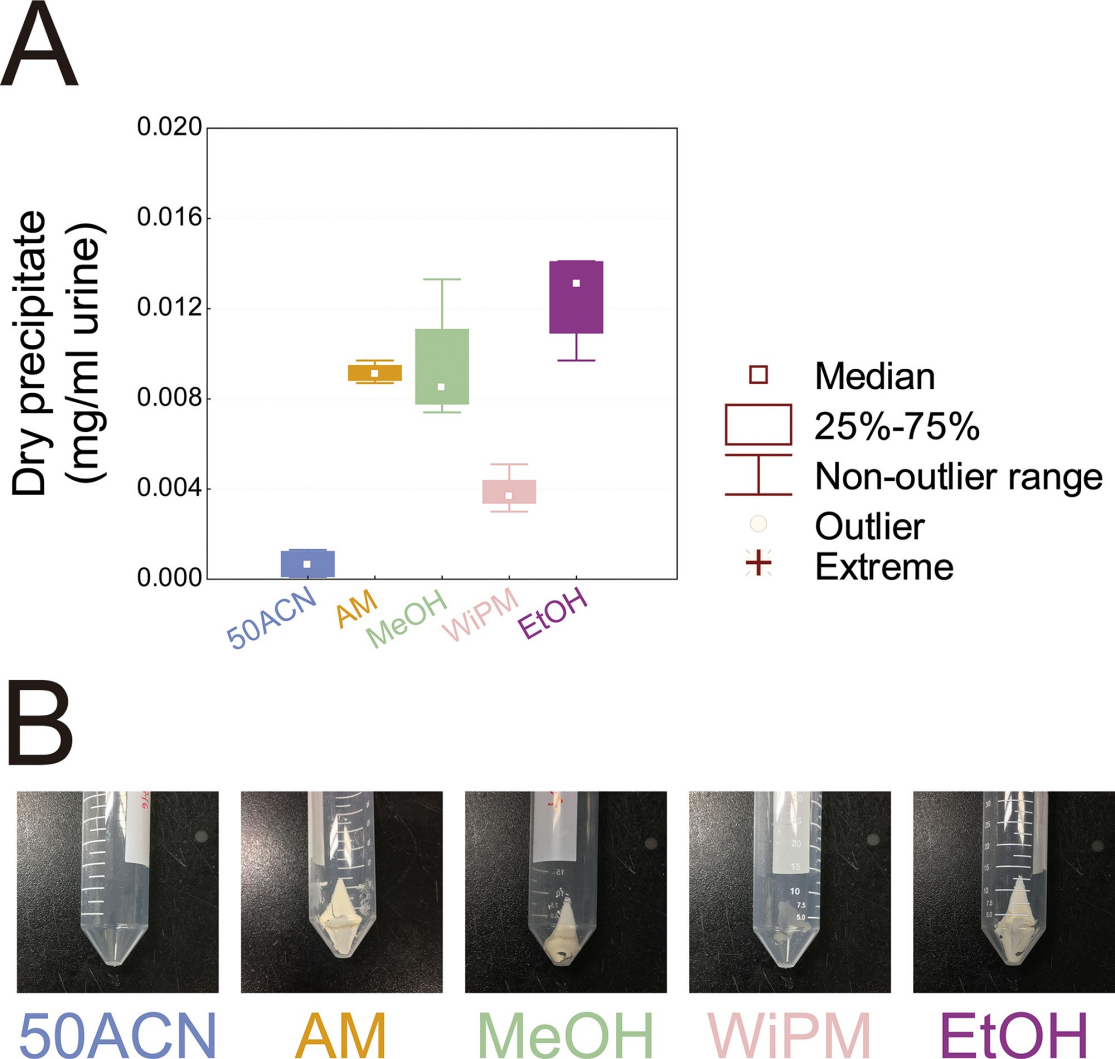
A comparison of extraction solvents on obtaining precipitates from urine samples. (A) Box and whisker plots of the amounts and (B) the photographs of dried precipitates. Thermally not treated pooled samples of urine were extracted with acetonitrile–water (50ACN; 1:1, v/v), formic acid–methanol (AM; 0.125:99.875, v/v), pure methanol (MeOH), water–isopropanol–methanol mixture (WiPM; 2:2:5, v/v/v), or pure ethanol (EtOH) at −20°C, and the precipitates were centrifuged and dried.

To evaluate the capabilities of precipitation of DNA, RNA, and proteins among the five solvents during metabolite extraction, centrifuged precipitates were dried and weighed (Fig 7). EtOH showed the highest precipitation capability, and AM and MeOH showed the second highest precipitation capabilities. WiPM showed much lower precipitation capability than did EtOH, AM, and MeOH. 50ACN did not precipitate DNA, RNA, and proteins.

## Discussion

The urease pretreatment step included in the standard urinary metabolome sample preparation, which involves incubation of samples with urease, has been a controversial topic [18,19] and contradicts the general concept of standard metabolome sample preparation, which requires immediate arrest of metabolic and enzymatic reactions [10,13,14,16]. In the present study, we found that urease pretreatment significantly alters human urinary metabolite profiles. For the first time, it was revealed that both urease and endogenous urinary enzymes convert urinary metabolites, thus significantly altering urinary metabolite profiles, leading to misinterpretation of metabolic status. Metabolites contained in urease preparations were also found to contaminate urinary metabolome samples. To recommend an optimal extraction solvent for human urinary metabolome sample preparation, five extraction solvents were assessed next. Among them, AM was found to be the best solvent with the highest capability and reproducibility of metabolite extraction and showed good capacity for precipitating DNA, RNA, and proteins.

In this study, urease, which is added to degrade urea from urine, was found to enzymatically act not only on urea but also on other metabolites, thus altering metabolite profiles (Fig 1A and 1B). After urease pretreatment, the gender-discriminating capabilities of urinary metabolomics significantly decreased (Fig 2). As for the metabolite profile changes after urease pretreatment, others also observed this effect [18,19] but did not find possible causes. In this study, after thermally treated urine samples were incubation with urease, the abundance levels of 82 metabolites significantly changed (Fig 4). The abundance changes of these 82 metabolites were likely to be mainly caused by the action of urease. Ureases used for pretreatment are extracted from the jack bean and are known to degrade not only urea into ammonia and CO_2_ but also other metabolites such as formamide, acetamide, and *N*-methylurea [42]. Besides, urease may have shown enzymatic promiscuity while being incubated with urine, where promiscuity means the ability of an enzyme to catalyze alternative reactions other than its native reaction [43,44]. Among these 82 metabolites, 10 upregulated metabolites were also detected as the contaminants originating from Type 3 and Type 4 ureases; amounts of these metabolites increased as the loading amounts of ureases in methanol extraction increased (Fig 5). Therefore, the increases in the abundance of these 10 metabolites were likely to be caused by the contaminants in urease preparations, whereas abundance changes in the other 72 metabolites may be attributed to the activity of urease.

Similar to urease pretreatment groups, in thermally not treated urine samples, endogenous urinary enzymes were found to enzymatically act on many urinary metabolites (Fig 3A). After thermal treatment of urine samples for inactivation of urinary enzymes, significant changes in metabolite profile were not observed even after incubation with water (Fig 3B and S1A Fig). Although the thermal treatment resulted in changes in the metabolite concentration possibly due to the Millard reaction and redox reaction [45,46], it was confirmed that the same types of metabolites were retained over a certain concentration before and after the thermal treatment (S1B Fig). These results suggest that even without urease, incubation of urine samples allows endogenous urinary enzymes to act on urinary metabolites. Therefore, not only urease itself but also the incubation process needs to be regarded as one of the main factors altering urinary metabolite profiles. Others have also observed metabolite abundance changes after incubation of urinary metabolome samples with water [19], but they did not find possible causes of these changes. Thus, the changes in metabolite profiles and in abundance levels of urinary metabolites appear to be caused by both urease pretreatment and endogenous urinary enzymes.

Not only the activities of urease and endogenous urinary enzymes discussed above but also contaminants from urease preparations can cause alterations in metabolite abundance and profiles, thus leading to misinterpretation of metabolomic information. For example, in the gender-discriminating PLS-DA model of group UI, the VIP value of lactose (which was also found to be a contaminant derived from the stabilizing agent in urease [19,47]) was only 0.309 (Fig 2 and S5 Table). Nonetheless, in the gender-discriminating PLS-DA model of group DE, the VIP value of lactose was 0.862 (Fig 2E and 2F and S5 Table). The significantly lower value of VIP in group UI than in group DE was due to the lactose contaminant from urease. Urease pretreatment increased the abundance of lactose, resulting in a similarity in lactose abundance between male and female urine samples, thus decreasing the VIP value in group UI. This decreased VIP value of group UI eventually reduced the gender-discriminating ability of the PLS-DA model (Fig 2A and 2B) because of the decreased VIP value (S5 Table).

The high concentrations of urea in urine samples are typical [2]. Therefore, urease pretreatment for degrading urea has been the key prerequisite for chromatographic analysis on the basis of the following assumptions: the large amounts of urea can cause overlapping of the peak of urea with peaks of other metabolites in chromatograms [24–26]; can result in overloading of chromatographic columns [27]; and can cause incomplete derivatization [19,20], thus interfering with identification of metabolites. On the other hand, because mass spectrometry was developed to be combined with chromatography, the peak overlaps between urea and other metabolites stopped being a problem in metabolite identification and quantification performed by means of their specific mass spectra [18]. Furthermore, the column overloading caused by urea can be minimized by controlling the volume of urine samples [18]. Results of metabolite derivatization, such as trimethylsilylation of urinary metabolites, were not found to be affected by urea in urine samples [18,29]. Overall, urinary metabolomic analysis without urease pretreatment does not worsen identification of metabolites, but rather urease pretreatment introduces artefacts and misinterpretations into urinary metabolomic analysis. Therefore, urease pretreatment should be removed from the standard protocol.

In the present standard protocol for preparation of urinary metabolome samples [2], after urine is pretreated with urease, the urine sample is extracted with a solvent. Among the five tested extraction solvents in this study, AM was found to be the best solvent for urinary metabolite extraction, with the highest scores on metabolite extraction capability and reproducibility, along with a good ability to precipitate DNA, RNA, and proteins, which need to be removed to prevent interference with metabolite analysis [48]. Both AM and 50ACN showed the highest (similar) capabilities for extraction of urinary metabolites (Fig 6A), but only AM showed the highest performance on both extraction reproducibility and precipitation capability (Figs 6B and 7). The substance serving as the solvent for many metabolites in urine is water [27]. Therefore, most urinary metabolites are dissolved in water, and most of them are polar, and only a few, such as fatty acids bound to protein, are nonpolar [27,49]. Thus, organic solvents with high polarity are most suitable for the extraction of urinary metabolites. The polarity of the solvents at 25°C is as follows: acetonitrile, 0.460; ethanol, 0.654, isopropanol, 0.546; methanol, 0.762; and water, 1.000 [50]. Given that the extraction solvents used in this study are complex mixtures of the above solvents, the exact polarity of each extraction solvent is difficult to calculate. Nevertheless, owing to the high polarity of methanol and water, the approximate polarity values can be presumably compared as follows in these two groups, AM > MeOH > EtOH and 50ACN > WiPM. In this study, the metabolite extraction capabilities of the extraction solvents were ranked in the following order 50ACN ≈ AM > MeOH > WiPM > EtOH (Fig 6A); these capabilities, to a certain extent, correspond to their relative polarity. Therefore, 50ACN and AM were found to possess the highest capabilities for extraction of urinary metabolites in this study.

In the comparison of 50ACN and AM on extraction (Fig 6B) and precipitation capabilities (Fig 7), AM showed much higher performance than 50ACN. The precipitation capability of an extraction solvent is inversely related to the dielectric constant of this solvent; the higher the dielectric constant, the lower the ability to precipitate DNA, RNA, and proteins [51]. The dielectric constants of the solvents are as follows: acetonitrile, 37.50; ethanol, 24.30; isopropanol, 18.30; methanol, 32.63; and water, 80.37 at 25°C [52]. Among the extraction solvents, acetonitrile has the lowest polarity but possesses the second highest dielectric constant. Thus, owing to the low precipitation capability of 50ACN, high-molecular-weight impurities dissolved in the solvent could be partially derivatized, thus resulting in irregular increases of peak intensities (Fig 7) [46]. This phenomenon explains the irregularly high intensities of amines and amino acids—the main components of DNA, RNA, and proteins—in 50ACN (Fig 6A). In contrast, the dielectric constant of acidified methanol is lower than that of 50ACN, causing AM to show a high precipitation capability (Fig 7) and extraction reproducibility (Fig 6B). Thus, owing to the high polarity and low dielectric constant of methanol, AM is the most suitable solvent for the extraction of urinary metabolites.

In conclusion, for accurate and reliable analysis of urinary metabolites, we propose to remove the controversial urease pretreatment step from the current standard urine metabolome preparation protocol, which was established for a chromatographic system a long time ago and has been used until today. In addition, we recommend AM as the ideal solvent for urinary metabolite extraction.

## Supporting information

S1 Fig PCA scores plots (A) and loading plots (B) of metabolite profiles for 113 metabolites identified in urinary metabolome samples obtained from 31 pooled male urine samples and 37 pooled female urine samples by the following different pretreatment methods. Group NT&WI, thermally not treated, incubated with water without urease, and extracted with pure methanol; group NT&DE, thermally not treated and extracted with pure methanol without urease pretreatment; group TT&WI, thermally treated, incubated with water without urease, and extracted with pure methanol; group TT&DE, thermally treated and extracted with pure methanol without urease pretreatment.
(TIF)

S2 Fig PCA score plots (A) and loading plots (B) of metabolite profiles of 113 metabolites identified in urinary metabolome samples obtained from 31 pooled and thermally treated male urine samples and 37 pooled and thermally treated female urine samples by the following different pretreatment methods. Group TT&UI, thermally treated, incubated with urease, and extracted with pure methanol; group TT&WI, thermally treated, incubated with water without urease, and extracted with pure methanol; group TT&DE, thermally treated and extracted with pure methanol without urease pretreatment.
(TIF)

S1 Table Characteristics of 68 healthy volunteers. (XLSX)

S2 Table Metabolome data analyzed by GC/TOF–MS. (XLSX)

S3 Table Identified 107 metabolites in 68 individual human urine samples. (XLSX)

S4 Table The Kruskal-Wallis test with post-hoc U test for the abundance comparison of metabolites identified from pooled urine samples pretreated by using the following different methods. UI, urine samples, incubated with urease and extracted with pure methanol; WI, incubated with water without urease and extracted with pure methanol; DE, extracted with pure methanol without urease pretreatment.
(XLSX)

S5 Table VIP values of 106 metabolites in PLS-DA models. (XLSX)

S6 Table Identified 113 metabolites in pooled urine samples. (XLSX)

S7 Table Non-parametric paired W test and U test for the comparisons of different urine pretreatment methods. NT&WI, pooled urine samples, thermally not treated, incubated with water without urease and then extracted with a solvent, pure methanol; NT&DE, pooled urine samples, thermally not treated and directly extracted with the solvent without urease pretreatment; TT&UI, pooled urine samples, thermally treated, incubated with urease and extracted with the solvent; TT&WI, pooled urine samples, thermally treated, incubated with water without urease and then extracted with the solvent; TT&DE, pooled urine samples, thermally treated and directly extracted with the solvent without urease pretreatment.
(XLSX)

S8 Table A. The Kruskal-Wallis test with post-hoc U test for the comparison of 12 metabolites depending on enzyme loading units of the Type 3 urease. B. The Kruskal-Wallis test with post-hoc U test for the comparison of 11 metabolites depending on enzyme loading units of the Type 4 urease. (XLSX)
