# Supplementary figures and images for: Systematic re-evaluation of the long-used standard protocol of urease-dependent metabolome sample preparation
Source: PLoS One. 2020 Mar 17;15(3):e0230072. doi: 10.1371/journal.pone.0230072 (PMC7077817; doi:10.1371/journal.pone.0230072)

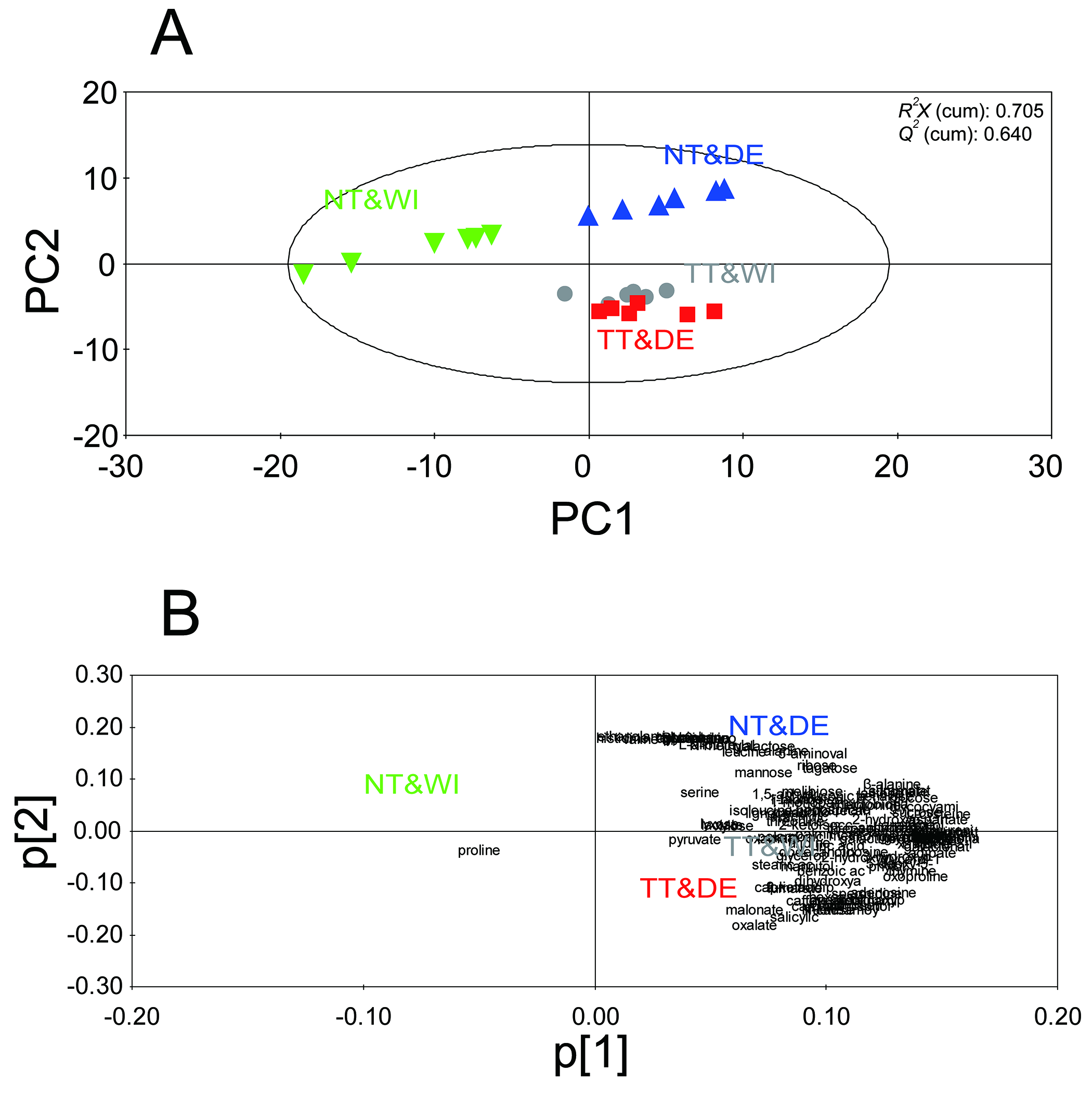

Supplement: S1 Fig — Group NT&WI, thermally not treated, incubated with water without urease, and extracted with pure methanol; group NT&DE, thermally not treated and extracted with pure methanol without urease pretreatment; group TT&WI, thermally treated, incubated with water without urease, and extracted with pure methanol; group TT&DE, thermally treated and extracted with pure methanol without urease pretreatment. (TIF) [file pone.0230072.s001.tif]

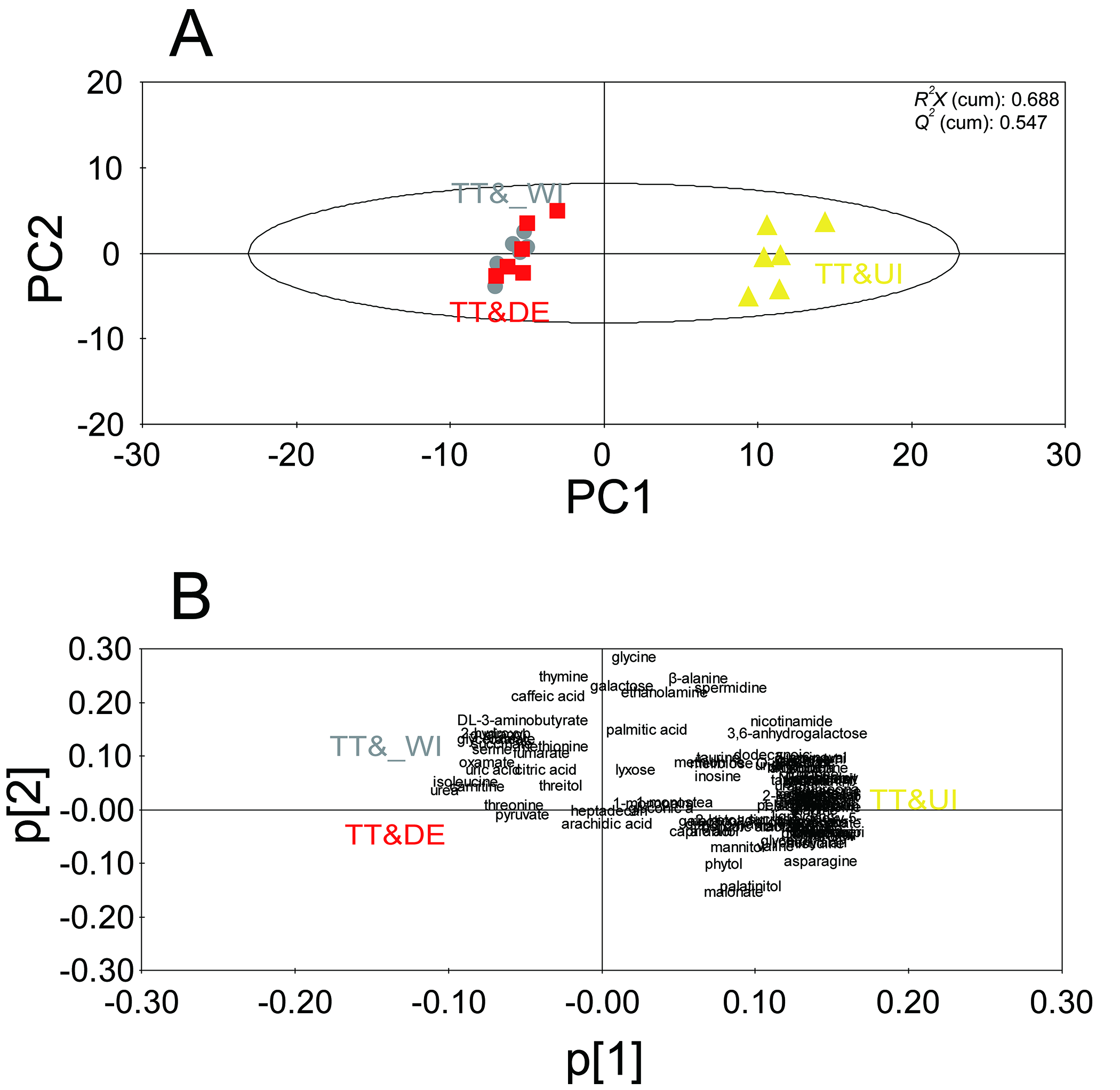

Supplement: S2 Fig — Group TT&UI, thermally treated, incubated with urease, and extracted with pure methanol; group TT&WI, thermally treated, incubated with water without urease, and extracted with pure methanol; group TT&DE, thermally treated and extracted with pure methanol without urease pretreatment. (TIF) [file pone.0230072.s002.tif]
